# Supplementary material for: Engineered Human Monoclonal scFv to Receptor Binding Domain of Ebolavirus
Source: Vaccines (Basel). 2021 May 4;9(5):457. doi: 10.3390/vaccines9050457 (PMC8147973; doi:10.3390/vaccines9050457)
Supplement: Supplementary file 1 [file vaccines-09-00457-s001.zip › vaccines-1189752-supplementary.pdf]

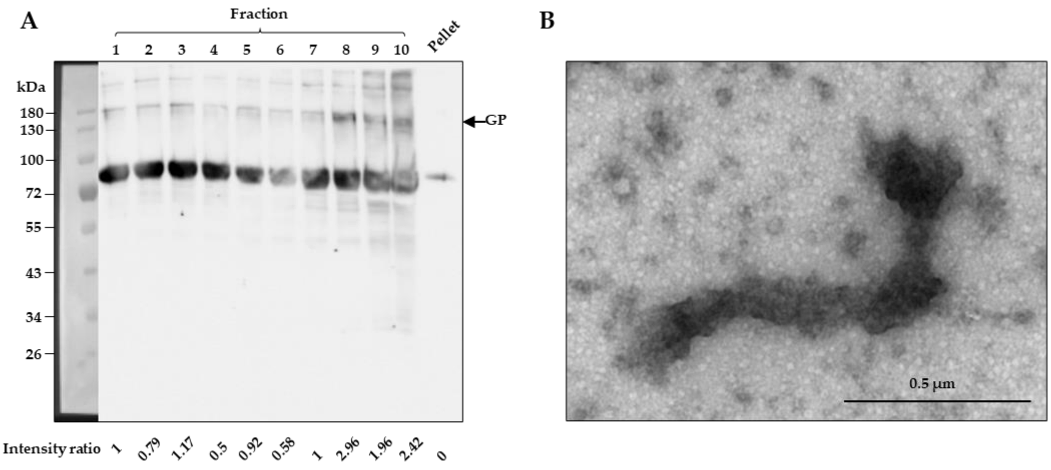

Figure S1. Production and characterization of VLP-bla. (**A**): Western blot pattern of density gradient-purified fractions of VLP sample probed with anti-GP antibodies. The first lane and the numbers at the left are protein marker and protein molecular masses in kDa, respectively. The numbers under lanes 1-10 indicate the ratio of the intensity of the expected band (arrow) over the intensity of the band in lane 1 as determined by densitometric analysis. (**B**): Representative image of the Ebola VLP revealed by negative staining and transmission electron microscopy.

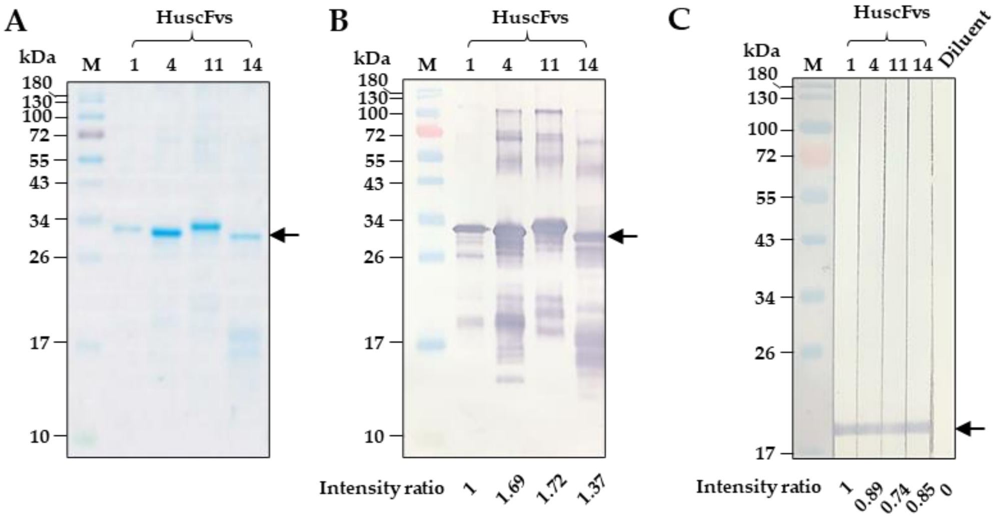

Figure S2. Production of non-cell-penetrable HuscFvs. (**A**): SDS-PAGE and CBB stained HuscFvs of *E. coli* clones 1, 4, 11 and 14 at ~26-34 kDa (arrow). (**B**): Western blot patterns of HuscFvs of *E. coli* clones 1, 4, 11 and 14 (arrow) as detected by anti-6x His antibody. (**C**): HuscFv-rRBD reactive bands (arrow) in Western blot analysis. Lanes M and numbers on the left of all blocks are protein molecular weight marker and protein molecular masses in kDa, respectively. The numbers under lanes 1, 4, 11 and 14 indicate the ratio of the intensity of the expected band (arrow) over the intensity of the band in lane 1 as determined by densitometric analysis.

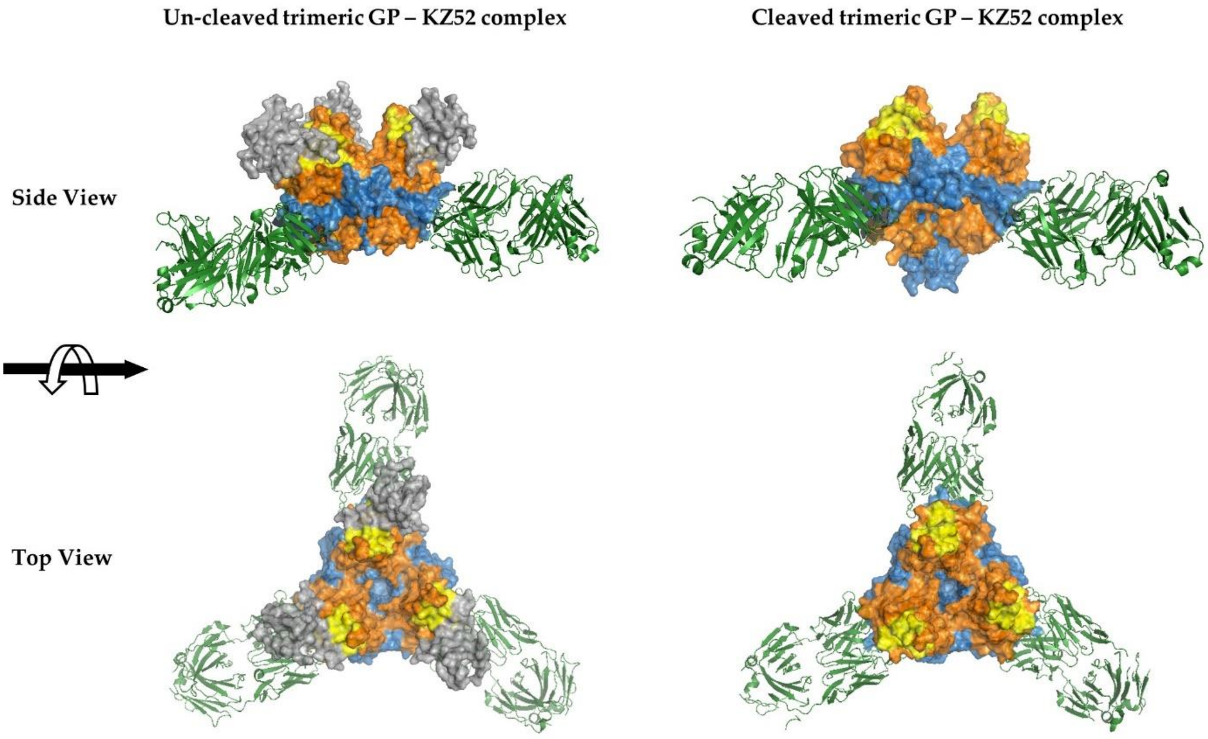

Figure S3. Crystal structure of GP: Published crystal structure of antibody KZ52 in complex with uncleaved trimeric GP (left panel, PDB 3CSY [3]) or cleaved trimeric GP (right panel, PDB 5HJ3 [19]) are shown as side view (upper panel) or top view (lower panel). KZ52 was colored in dark green. GP was colored as in Figure 5 along with glycan cap (GC) in grey.

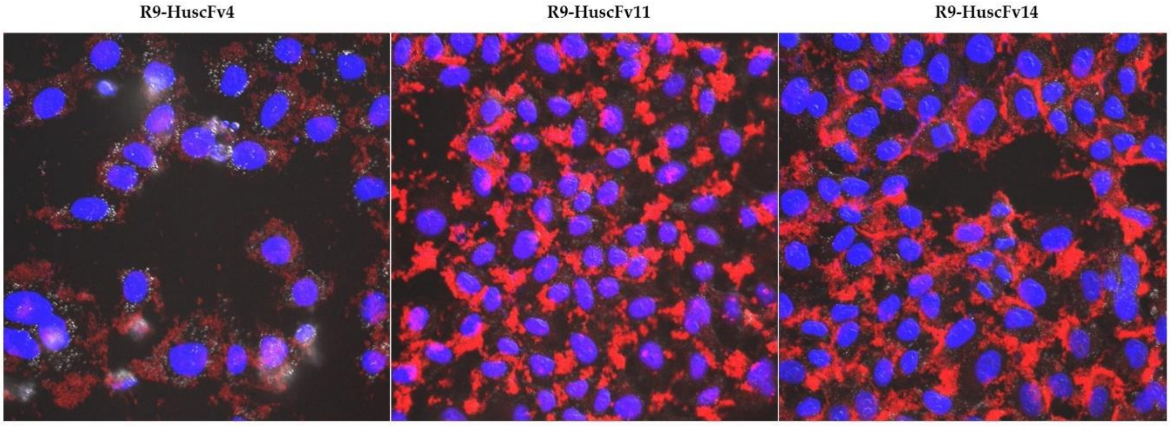

Figure S4. Patterns of intracellular distribution of R9-HuscFvs: HEK293T cells were incubated with 30 μg/mL of R9-HuscFv4 (left panel), R9-HuscFv11 (middle panel) or R9-HuscFv14 (right panel) for 24 h and the cells were observed by confocal microscopy (60× objective lens). The R9-HuscFvs appear red in cytoplasm; the nuclei are blue by DAPI staining.

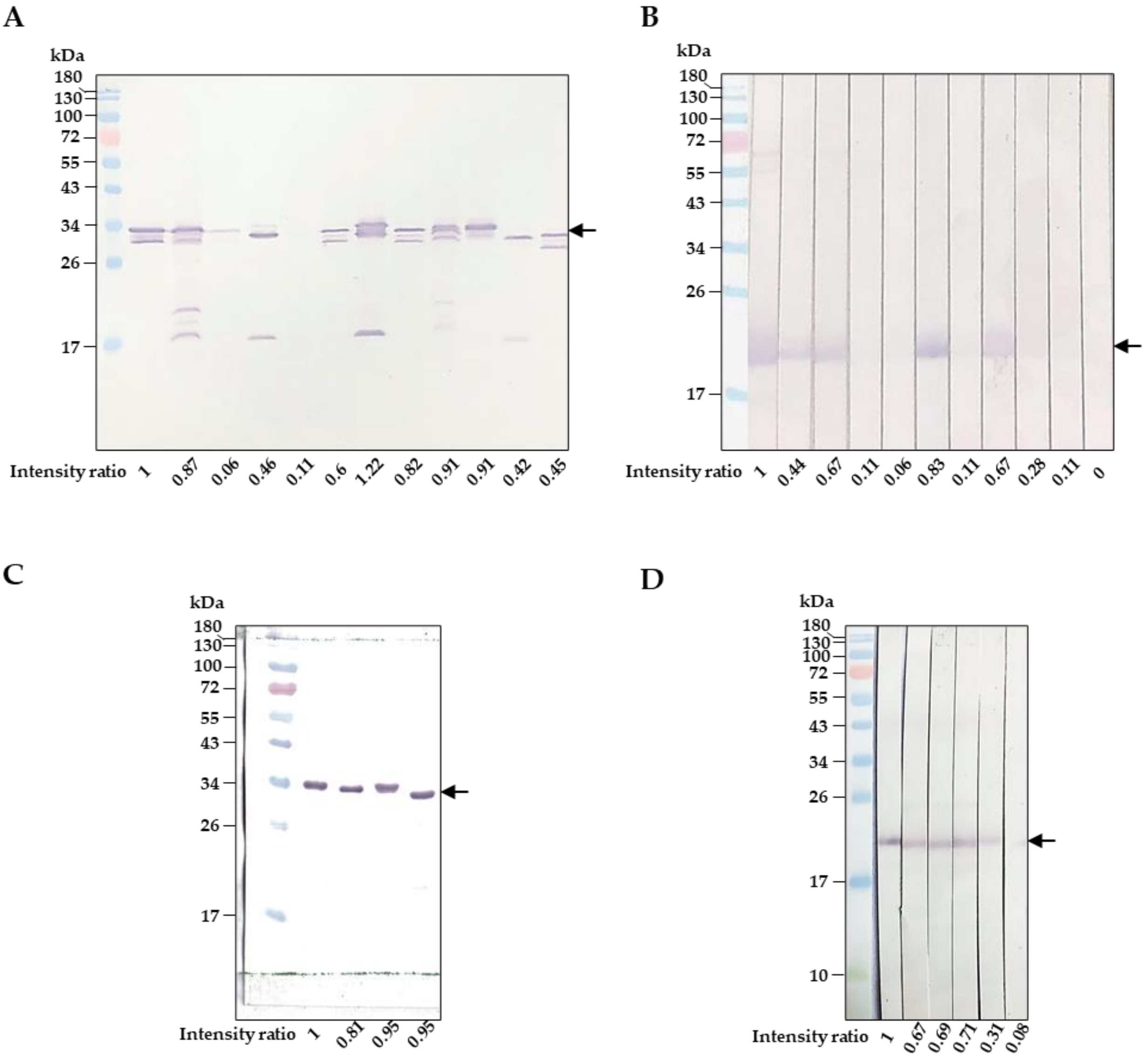

Figure S5. Full, uncropped Western blot images that correspond to Figure 1D (**A**), Figure 1F (**B**), Figure 2B (**C**) and Figure 2D (**D**). The first lanes and numbers at the left of all blocks are protein marker and protein molecular masses in kDa, respectively. The number under each lane indicates the ratio of the intensity of the expected band (arrow) over the intensity of the band in the second lanes (from left) determined by densitometric analysis.
